# Mammary carcinoma in a male cat following long-term medroxyprogesterone acetate treatment: case report and review of the literature

**DOI:** 10.1007/s11259-024-10553-3

**Published:** 2024-09-28

**Authors:** Adina Mihaela Pîrvu, Andrea Cappelleri, Laura Sala, Barbara Banco, Chiara Giudice, Damiano Stefanello, Manuella Militaru, Valeria Grieco

**Affiliations:** 1 Pathological Anatomy Department, University of Agronomic Sciences and Veterinary Medicine of Bucharest, Bucharest, Romania; 2 Department of Veterinary Medicine and Animal Sciences, University of Milan, Lodi, Italy; 3 Mouse and Animal Pathology Laboratory (MAPLab), UniMi Foundation, Milan, Italy; 4 MYLAV La Vallonea Veterinary Diagnostic Laboratory, Rho, Italy

**Keywords:** Cat, Mammary gland, Carcinoma, Histology, Medroxyprogesterone acetate, Metastases

## Abstract

In male cats, as in men, mammary carcinomas are rarely reported. However, like in females, hormonal therapy is a significant risk factor. This study reports the case of an 11-year-old male cat with multiple mammary tumours and a history of long-term medroxyprogesterone acetate therapy for the suppression of sexual behaviour, along with a brief review of the literature. Complete surgical removal of the right mammary chain and the ipsilateral inguinal lymph nodes was performed, and all tissues were submitted for histology. Histological examination revealed the presence of a tumour in the third and fourth mammary glands, consisting of neoplastic cells arranged in various structures, including tubulopapillary and tubular structures, sometimes cystically dilated, and solid areas. The inguinal lymph nodes were also involved. The morphology was consistent with a diagnosis of mammary carcinoma, tubulopapillary type, with nodal metastases. Immunohistochemistry revealed that tumour cells were positive for cytokeratin (clones AE1/AE3), while stromal cells were positive for vimentin (clone V9). The proliferation marker Ki-67, evaluated on both the primary tumour and the nodal metastases, was strongly expressed in the nuclei of neoplastic cells, with a Ki-67 proliferation index of 8.9% and 20% for the primary tumour and the metastases, respectively. This case highlights the importance of considering the possibility of malignant mammary tumours not only in female but also in male cats with a history of long-term hormonal treatment for suppression of sexual behaviour.

## Background

Breast cancer is the most frequently diagnosed cancer in women worldwide (Łukasiewicz et al. 2021), while male breast cancer (MBC) is relatively rare, with men accounting for only 1% of breast cancer patients (Yalaza et al. 2016; Giordano 2018; Zheng and Leone 2022). Similarly, mammary tumours are the third most common type of neoplasia in female cats, while they are very rare in males, accounting for approximately 1–5% of diagnosed mammary carcinomas in felines (Skorupski et al. 2005; Jacobs et al. 2010). However, in both humans and cats, mammary carcinomas in males share many clinical similarities with their female counterparts, with hormonal treatment or imbalance being a major risk factor (Skorupski et al. 2005; Yalaza et al. 2016; Giordano 2018; Łukasiewicz et al. 2021; Zheng and Leone 2022). While early surgical spaying of cats has proven to be protective, several studies indicate that chemical spaying through exogenous administration of progestogens represents a major risk factor in the development of these tumours in cats of both sexes (Skorupski et al. 2005; Jacobs et al. 2010; Spugnini et al. 2010; Gregório et al. 2012).

In the present report, we describe the histological and immunohistochemical features of a mammary carcinoma with lymph node metastases in a male cat with a history of long-term treatment with medroxyprogesterone acetate for the suppression of sexual behaviour.

## Case presentation

An 11-year-old male Domestic Shorthair cat was referred to the Veterinary Teaching Hospital of the University of Milan for multiple mammary nodules. The owner reported that the cat was cryptorchid and that the descended testis had been removed at an early age, while the undescended one was not found until 1.5 years before the clinical examination, when it was finally removed in a second surgery.

After the first orchiectomy, for suppression of male behaviour caused by the presence of the undescended testis, the cat received three subcutaneous injections per year of medroxyprogesterone acetate (50 mg/ml) over an eight-year period. The treatment was discontinued after the removal of the undescended testis. The cat was regularly vaccinated and otherwise healthy. The clinical examination identified multiple subcutaneous nodules (ranging from 0.3 to > 3 cm in diameter), non-painful and movable in the right third and fourth mammary glands. On palpation, no inguinal lymphadenomegaly was noted, and the physical examination was otherwise unremarkable. The nodules were sampled for cytology, and a diagnosis of carcinoma was achieved. On clinician recommendation, the owner decided to proceed with the unilateral mastectomy of the right chain. Before surgery, thoracic radiographs and abdominal ultrasonography were performed to exclude distant metastases, and both exams were negative. Laboratory tests (blood count, biochemistry profile, and urine analysis) were within the normal range, except for mild thrombocytopenia and lymphopenia. Therefore, a unilateral mastectomy of the right chain with the removal of the ipsilateral inguinal lymph nodes was performed. The tissues removed were sent to the Pathology Unit for histological examination.

Grossly, the first thoracic mammary gland was apparently normal; the second was characterised by multiple small cystic lesions, and the third and fourth mammary glands were expanded by a white and solid plaque-like lesion of approximately 4 cm in diameter. Multifocal whitish lesions were noted within the inguinal lymph nodes as well. Based on gross findings, differential diagnoses included neoplastic lesions (most likely mammary carcinoma or adenocarcinoma) or fibroadenomatous hyperplasia.

**Fig. 1:**
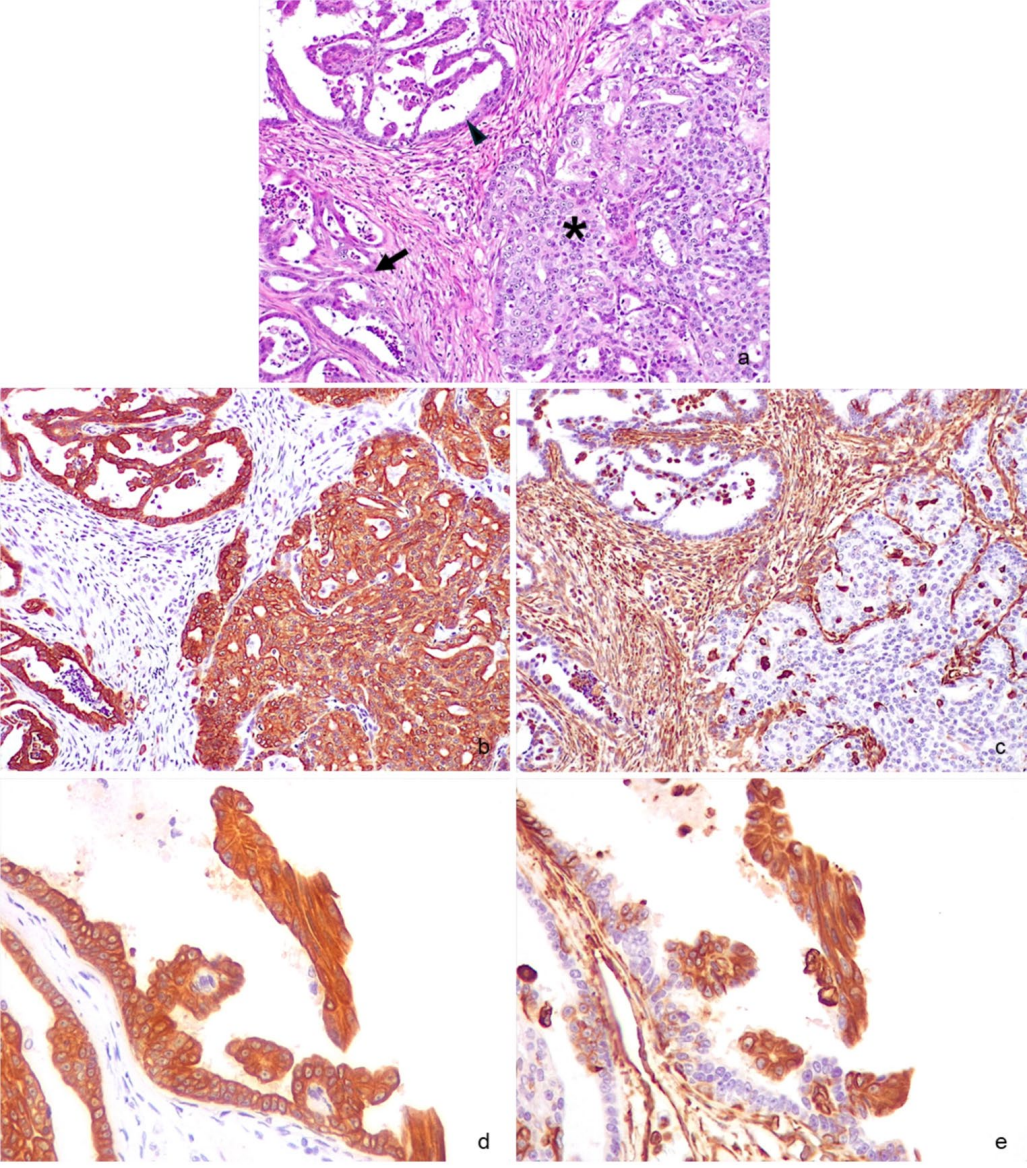
Mammary gland. **a** Tubulopapillary carcinoma with marked desmoplasia. Note the presence of multiple patterns: solid (*asterisk*), tubular (*arrow*), and tubulopapillary (*arrowhead*); H&E; 200x. **b** Same field. Strong cytoplasmic labelling of neoplastic cells for cytokeratin AE1/AE3; IHC; 200x. **c** Same field. Vimentin V9 strongly labels the cytoplasm of fibroblasts immersed in desmoplastic collagen; IHC; 200x. **d**, **e** Note the presence of groups of sloughed neoplastic cells positive for both cytokeratin AE1/AE3 (d) and vimentin V9 (e), suggestive of epithelial-to-mesenchymal transition; IHC; 400x

Tissue samples from all the mammary glands and the lymph nodes were collected, fixed in 10% neutral buffered formalin for three days, and then trimmed and routinely processed for histology. Briefly, samples were dehydrated through graded alcohols, clarified in xylene, and embedded in paraffin. Four-micrometre-thick sections were obtained from the paraffin blocks and stained with haematoxylin and eosin (H&E). Histologically, the first mammary gland was characterised by moderate lobular hyperplasia, multifocal ductal cysts, and duct ectasia. The second mammary gland was characterised by lobular mammary hyperplasia and cysts filled with abundant proteinaceous material consistent with mammary secretion; the third and fourth mammary glands were entirely replaced by a highly cellular, unencapsulated, multinodular, infiltrative neoplasm, with multifocal large necrotic areas. Neoplastic cells were cuboidal to columnar, ranging from 10 to 15 micrometres in diameter, with indistinct cell borders, a moderate amount of eosinophilic cytoplasm, and a large vesicular round to oval nucleus with a prominent centrally located nucleolus. Neoplastic cells were arranged in tubular and papillary structures, with occasional areas of solid growth, supported and separated by dense fibrovascular and desmoplastic stroma (Fig. 1a). Tubular lumina contained necrotic debris, sloughed epithelial cells admixed with secretion, and scattered neutrophils and foamy macrophages. Peri- and intraluminal lymphoplasmacytic aggregates were also observed. Lymphatic vessels were often ectatic, and multifocally contained solid aggregates of neoplastic cells (emboli).

To better characterise the neoplasia, serial sections were obtained and automatically stained for immunohistochemistry (IHC) with the avidin-biotin-peroxidase complex method using the Thermo Scientific™ Autostainer 480 S. The primary antibodies used are listed in Table 1. For all markers, internal positive controls were used, while sections of the samples without primary antibodies were used as negative controls. For double immunofluorescence (IF), the same mouse anti-vimentin antibody (1:1500) used for IHC was applied, along with a goat polyclonal anti-Iba-1 antibody (1:1000; Novus Biologicals, Centennial, CO, USA). For both markers, internal positive controls were used.

**Table 1:** Primary antibodies for immunohistochemistry (IHC)

IHC Marker	Antigen retrieval	Primary antibody	Code	Species
Cytokeratin (AE1/AE3)	HIER; Buffer H pH 9*	Monoclonal, dilution: 1:1000; Agilent Dako, Santa Clara, CA, USA	M3515	Mouse
Ki-67 (MIB-1)	HIER; Buffer H pH 9	Monoclonal, dilution: 1:1000; Agilent Dako, Santa Clara, CA, USA	M7240	Mouse
Vimentin (V9)	HIER; Buffer H pH 9	Monoclonal, dilution: 1:3000; Thermo Fisher Scientific, Waltham, MA, USA	MA5-11883	Mouse

*Dewax and HIER Buffer H (Epredia, Kalamazoo, MI, USA)

**Fig. 2:**
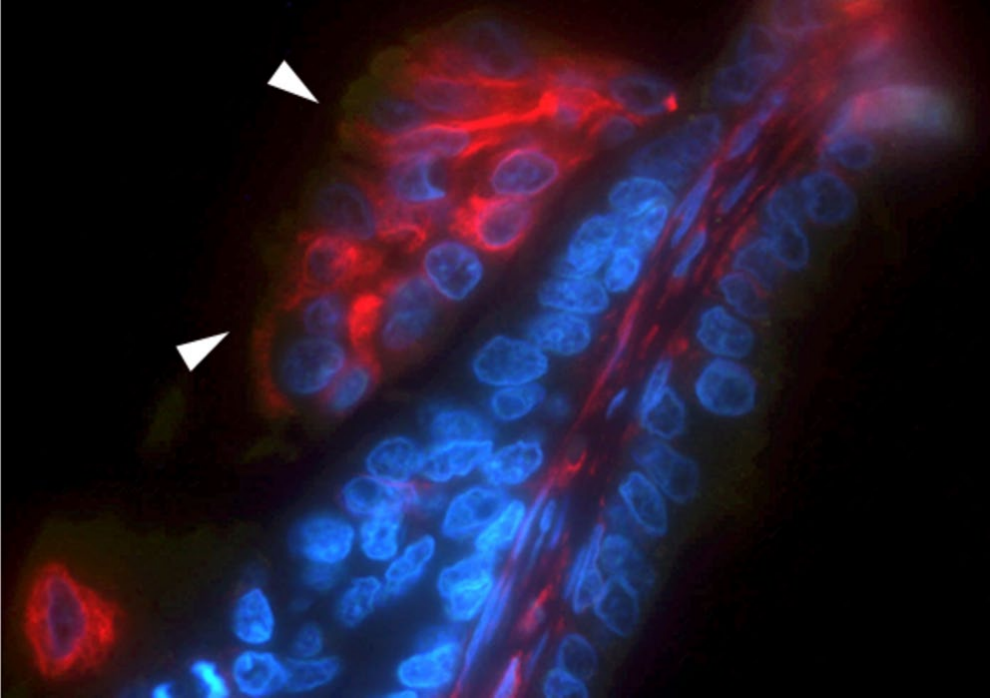
Mammary gland. Tumour papilla lined by neoplastic epithelial cells. Neoplastic cells attached to the stalk of the papilla are negative for vimentin (*red*), while the group of sloughed cells (*white arrowheads*) are strongly positive for the same marker. Neoplastic cells are negative for Iba-1 (*green*). Nuclei are stained with DAPI (*blue*). Vimentin V9 + Iba-1 + DAPI (merged); IF; 1000x

Neoplastic cells lining the tubules and papillae displayed strong cytoplasmic immunolabelling for cytokeratins (Fig. 1b), while vimentin was positive in the cytoplasm of stromal and desmoplastic fibroblasts (Fig. 1c). Moreover, some neoplastic cells sloughed in the lumen of the ducts, either isolated or in small groups, were variably positive for both markers (Fig. 1d, e). Confirmation that sloughed cells were actually neoplastic and not macrophages came also from the double IF (Fig. 2).

**Fig. 3:**
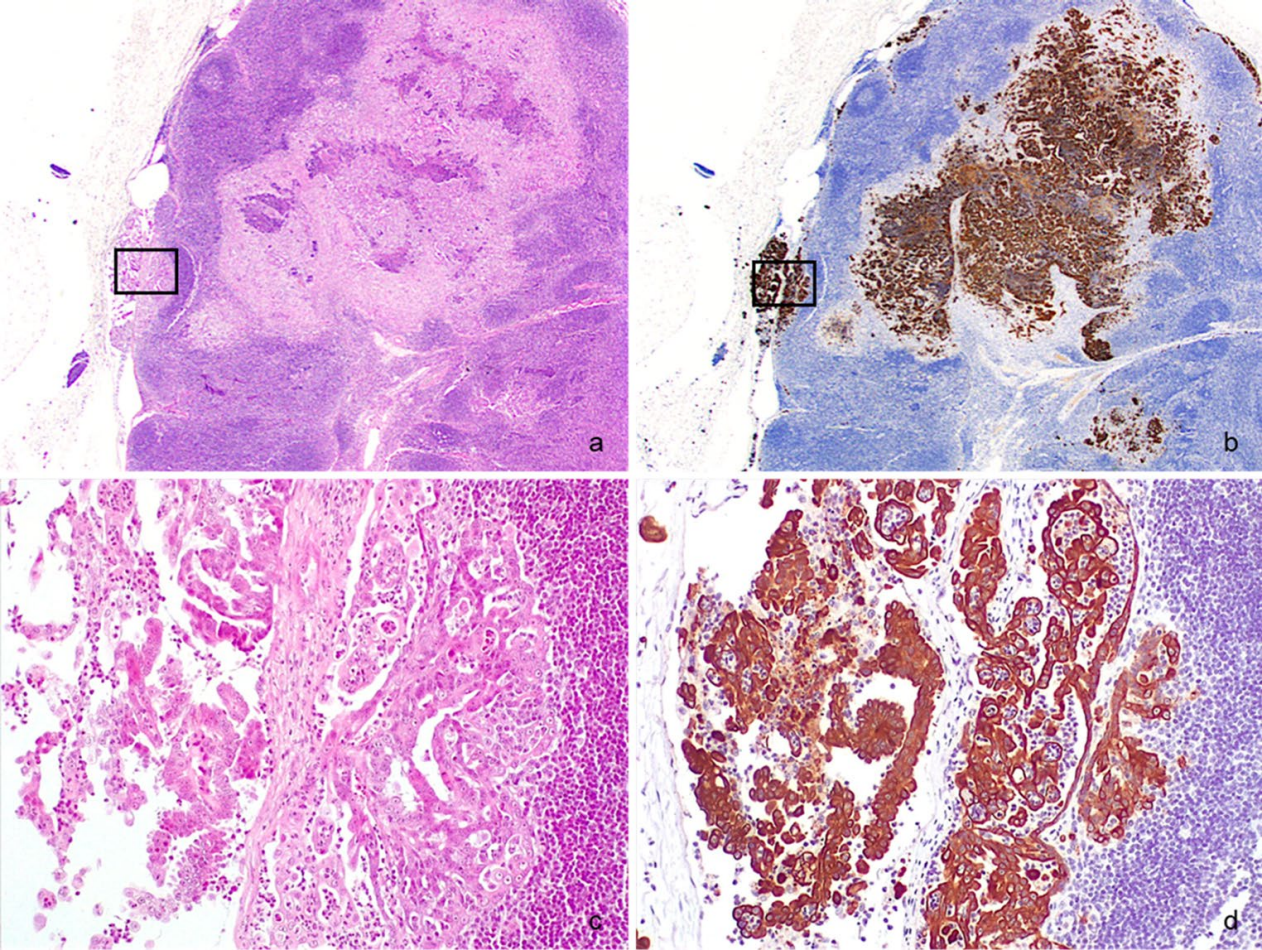
Inguinal lymph node. **a** The parenchyma is expanded and partially replaced by a tumour; H&E; 25x. **b** Same field. Strong cytoplasmic labelling of neoplastic cells for cytokeratin AE1/AE3; IHC; 25x. **c** Higher magnification of *square region* in (a). The subcapsular sinus is dilated and the cortex invaded by a carcinoma with tubulopapillary growth; H&E; 200x. **d** Higher magnification of *square region* in (b). Strong cytoplasmic labelling of neoplastic cells for cytokeratin AE1/AE3; IHC; 200x

The nodal parenchyma was extensively replaced by multiple metastases, morphologically consistent with the neoplasia previously described (Fig. 3a, c). Follicular hyperplasia was also present. Immunolabelling for cytokeratins revealed neoplastic cells mainly concentrated within the subcapsular and medullary sinuses and within the medullary cords (Fig. 3b, d). Based on histomorphology and IHC, a diagnosis of tubulopapillary mammary carcinoma with inguinal lymph node metastases was made.

**Fig. 4:**
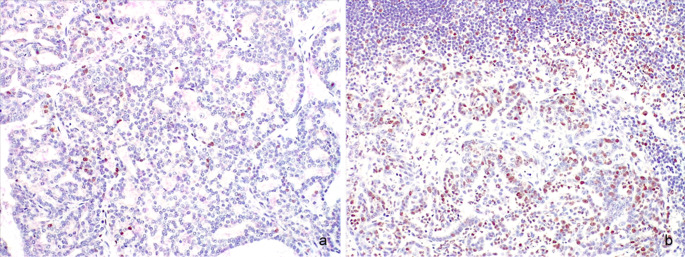
Mammary gland (**a**) and inguinal lymph node (**b**). Strong nuclear staining for Ki-67 in the neoplastic cells. Note the higher number of neoplastic positive cells in the nodal metastasis compared with the primary tumour; IHC; 200x

The proliferative index measured by Ki-67 was also evaluated on both the primary tumour and the nodal metastases, as previously described (Castagnaro et al. 1998; Millanta et al. 2002; Soares et al. 2016). Briefly, the Ki-67 proliferation index was determined by assessing the percentage of positively stained tumour cell nuclei in 1000 tumour cells. Ki-67 was strongly expressed in the nuclei of neoplastic cells (Fig. 4a, b). The Ki-67 proliferation index was 89.4 (8.9%) and 200 (20%) for the primary tumour and the metastases, respectively.

The adjuvant chemotherapy was proposed, but the owner refused it. One year after the diagnosis, the owner reported that the cat was humanely euthanised due to progressive respiratory distress and cachexia. Based on the owner’s information, metastatic spread to other organs, particularly the lungs, may be hypothesised.

### Discussion and conclusions

Male breast cancer (MBC) is relatively rare in human medicine. On average, men are diagnosed at a later age compared to women (the median age being 65 years) and at a more advanced stage (Yalaza et al. 2016; Giordano 2018; Zheng and Leone 2022). They also have a lower overall survival rate. Due to low public awareness and the absence of screening programmes, men are more likely than women to present with regional nodal metastases (Giordano 2018). Risk factors include hormone imbalance, occupational and environmental exposures, and genetic factors such as family history, BRCA2 mutations, or Klinefelter syndrome (Yalaza et al. 2016; Giordano 2018; Zheng and Leone 2022). Testicular abnormalities (e.g., cryptorchidism, orchitis, orchidectomy) may also be associated with MBC (Yalaza et al. 2016; Giordano 2018; Zheng and Leone 2022). High oestrogen levels play an important role in MBC development, as do obesity and cirrhosis, which can both lead to increased oestrogen levels (Yalaza et al. 2016; Giordano 2018; Zheng and Leone 2022). Interestingly, men have a poorer prognosis despite having a high rate of hormone receptor immunohistochemical expression, which in women is associated with a more favourable outcome (Giordano 2018; Zheng and Leone 2022).

Similar to humans, mammary carcinoma is rare in male cats (Hayes 1977; Skorupski et al. 2005; Gregório et al. 2012). Mammary tumours in cats are usually diagnosed between 10 and 12 years of age, are highly aggressive, and frequently metastasise to regional lymph nodes (Goldschmidt et al. 2016). Regarding breed predisposition, they seem to be more common in Siamese cats (Goldschmidt et al. 2016). Only a few studies on mammary tumours in male cats are available in the literature (Skorupski et al. 2005; Baștan et al. 2007; Loukopoulos et al. 2007; Pires et al. 2008; Jacobs et al. 2010; Gregório et al. 2012), and they indicate similar behaviour to tumours in female cats. The mean age at diagnosis (11.5 years) is also similar to females (Skorupski et al. 2005; Loukopoulos et al. 2007; Gregório et al. 2012; Connah 2016; Tay et al. 2024). The overall median survival time (calculated on a caseload of 27 cats) is 344 days for male cats with mammary carcinoma, which is greater than the 7–10 months reported for female cats (Skorupski et al. 2005). Reported breeds of male cats with mammary carcinoma are Domestic short-hair, Siamese, Domestic long-hair, and Persian (Skorupski et al. 2005; Baștan et al. 2007; Loukopoulos et al. 2007; Gregório et al. 2012; Tay et al. 2024).

In men, ductal carcinoma is the most prevalent histological subtype (Yalaza et al. 2016; Giordano 2018; Zheng and Leone 2022). In male cats, the few cases described in the literature are either ductal adenocarcinomas or invasive micropapillary carcinomas (Pires et al. 2008; Gregório et al. 2012; Connah 2016; Tay et al. 2024). In our study, a mixed pattern was observed, with some areas consistent with a tubulopapillary type and others encompassing more solid areas. However, the paucity of cases described so far makes it difficult to determine whether there is a characteristic histological subtype for male cats’ mammary carcinomas, and studies on larger caseloads are needed for confirmation. Nonetheless, it is known that feline invasive micropapillary carcinoma is a biologically aggressive tumour with decreased survival due to its tendency to invade lymphatic vessels and metastasise (Seixas et al. 2007). Tumour size (> 3 cm) and lymphatic invasion are, to date, the only factors negatively correlated with the survival of male cats with mammary carcinoma (Skorupski et al. 2005). The case described herein had both lymphatic invasion and a tumour size > 3 cm. However, the cat survived up to one year after diagnosis without adjuvant antineoplastic chemotherapy, which is longer than what is reported in the literature for tumours with these features (Skorupski et al. 2005). On the other hand, this time span matches the overall median survival time for male cats with mammary carcinoma (Skorupski et al. 2005). Grading has not been performed in previously reported cases of mammary carcinoma in male cats (Skorupski et al. 2005; Pires et al. 2008; Jacobs et al. 2010; Gregório et al. 2012). While two different grading systems have been proposed for feline mammary carcinomas, there is no definitive consensus on which system should be used (Zappulli et al. 2019; Avallone et al. 2021). The proliferative index measured by Ki-67 was evaluated in a single case of male feline mammary carcinoma (Gregório et al. 2012). To date, no consensus or standardisation has been reached regarding Ki-67 index cut-offs for feline mammary carcinomas (Castagnaro et al. 1998; Millanta et al. 2002; Zappulli et al. 2015; Soares et al. 2016). As a confirmation of the lack of consistency on this matter, the Ki-67 proliferation index in our case was below both the earliest cut-off proposed (25.2%) (Castagnaro et al. 1998), and the cut-off proposed later on (14%) (Soares et al. 2016). Interestingly, in agreement with the same study (Soares et al. 2016), the Ki-67 index in the case described herein was higher in the nodal metastases than in the primary tumour.

Moreover, in our case, a few neoplastic cells, especially those detached and free in the ducts, were positive for vimentin, which is suggestive of epithelial-to-mesenchymal transition (EMT). Since foamy macrophages are frequently found in mammary tumours admixed with sloughed neoplastic cells and secretion within ductal and tubular lumina (Zappulli et al. 2019), the possibility that a small number of these vimentin positive cells were macrophages cannot be completely excluded, especially for rounded and individualised cells. However, double IF revealed the neoplastic origin in the vast majority of these cells, further supporting the EMT transition. EMT is a well-described process that allows a polarised epithelial cell to assume a mesenchymal phenotype (Kalluri and Weinberg 2009; Nistico et al. 2012). This has been closely associated with cancer cell aggressiveness, invasiveness, and elevated resistance to apoptosis and has also been reported in feline mammary carcinomas (Sammarco et al. 2023). This feature correlates well with the presence of nodal metastases in our case.

The role of hormones in the development of feline mammary carcinoma remains central. Exogenous progestins have been shown to increase the risk of developing mammary tumours by 3.4 times in both female and male cats (Skorupski et al. 2005; Goldschmidt et al. 2016). Spayed females and intact or castrated males exposed to progestins are also at risk of developing mammary neoplasia (Hayden et al. 1989; Zappulli et al. 2019). The case reported herein had features consistent with those previously described in both female and male cats treated with progestin therapy (Skorupski et al. 2005; Jacobs et al. 2010). In the retrospective study by Skorupski and colleagues (Skorupski et al. 2005), one-third of the male cats with mammary carcinoma had a history of progestin therapy. Local and/or regional relapse was commonly reported (45%), and lymphatic invasion was identified in 44% of male cats. Similar results were also reported by Jacobs and colleagues (Jacobs et al. 2010), who described local recurrences despite radical mastectomies being performed (Jacobs et al. 2010).

Medroxyprogesterone acetate, also known as 17α-hydroxy-6α-methylprogesterone acetate, is a steroidal progestin, a synthetic variant of the human hormone progesterone. It is commonly given to female cats for reproductive control and to male cats as an alternative to gonadectomy for behavioural modification (urine spraying/marking, reducing sexual behaviour including mounting, mating, and aggression) (Hart 1980; Kutzler and Wood 2006) and eosinophilic and proliferative keratopathies (Spiess et al. 2009). The use of progestins as therapeutic agents for cats increases the possibility of serious side effects such as adrenal cortical suppression, glucose intolerance, hepatotoxicity, pyometra, mammary fibroadenomatous hyperplasia (Dorn et al. 1983; Hayden et al. 1989; MacDougall 2003; Torrigiani et al. 2022), and/or mammary carcinoma (Oen 1977; Tomlinson et al. 1984; Goldschmidt et al. 2016; Zappulli et al. 2019). The risk of these side effects is significantly higher in animals undergoing long-term (several years) treatment (Romagnoli and Concannon 2003). Dysplastic lesions of mammary tissue, such as fibroadenomatous change, may eventually undergo malignant transformation with continued progestin treatment (Mol et al. 1996; Loretti et al. 2005; Skorupski et al. 2005; Jacobs et al. 2010; Goldschmidt et al. 2016). We do not exclude such a situation for the present case, given that the first and second glands were affected by hyperplastic changes. A possible initial development of fibroadenomatous hyperplasia was considered, which could have represented a preneoplastic lesion.

Moreover, as in this case, the development of a mammary tumour may occur years after drug administration (Jacobs et al. 2010). Interestingly, wild felids treated with contraceptives for reversible reproductive control develop the same kind of lesions described in domestic cats (Kollias Jr 1988).

One study also reported mammary carcinoma in two male cats following contraceptive treatment with the steroid anti-androgen cyproterone acetate (Spugnini et al. 2010). The mechanism of action of this substance on male cat mammary tissue is unknown; however, the authors described a possible gestagenic effect, which might have contributed to the initial hyperplastic changes and secondary malignant transformation (Spugnini et al. 2010). The same substance has been reported as the cause of gynaecomastia in a male cat (Jelinek et al. 2007). Cyproterone acetate has occasionally been associated with the development of gynaecomastia and breast cancer in men treated for prostatic malignancy (d’Ancona and Debruyne 2005; Spugnini et al. 2010).

In this report, we described a case of mammary carcinoma with a histopathological diagnosis of metastasis in the regional inguinal lymph node in a male cat following several years of treatment with medroxyprogesterone acetate. While mammary carcinoma is still a rare entity in male cats, veterinary clinicians and pathologists should be aware of this possibility, especially in animals with a history of long-term hormonal treatment for the suppression of sexual behaviour. For this reason, surveillance for immediate and long-term adverse effects, as well as knowledge of the benefit-cost ratio of currently available contraceptives, is recommended.

## Data Availability

No datasets were generated or analysed during the current study.
